# ESTimating plant phylogeny: lessons from partitioning

**DOI:** 10.1186/1471-2148-6-48

**Published:** 2006-06-15

**Authors:** Jose EB de la Torre, Mary G Egan, Manpreet S Katari, Eric D Brenner, Dennis W Stevenson, Gloria M Coruzzi, Rob DeSalle

**Affiliations:** 1 Department of Biology, New York University, 100 Washington Sq East, New York NY 10003, USA; 2 American Museum of Natural History, Central Park West @79^th ^St., New York, NY 10024, USA; 3 New York Botanical Garden Bronx, 200th Street and Kazimiroff Boulevard, Bronx, NY 10458, USA

## Abstract

**Background:**

While Expressed Sequence Tags (ESTs) have proven a viable and efficient way to sample genomes, particularly those for which whole-genome sequencing is impractical, phylogenetic analysis using ESTs remains difficult. Sequencing errors and orthology determination are the major problems when using ESTs as a source of characters for systematics. Here we develop methods to incorporate EST sequence information in a simultaneous analysis framework to address controversial phylogenetic questions regarding the relationships among the major groups of seed plants. We use an automated, phylogenetically derived approach to orthology determination called OrthologID generate a phylogeny based on 43 process partitions, many of which are derived from ESTs, and examine several measures of support to assess the utility of EST data for phylogenies.

**Results:**

A maximum parsimony (MP) analysis resulted in a single tree with relatively high support at all nodes in the tree despite rampant conflict among trees generated from the separate analysis of individual partitions. In a comparison of broader-scale groupings based on cellular compartment (ie: chloroplast, mitochondrial or nuclear) or function, only the nuclear partition tree (based largely on EST data) was found to be topologically identical to the tree based on the simultaneous analysis of all data. Despite topological conflict among the broader-scale groupings examined, only the tree based on morphological data showed statistically significant differences.

**Conclusion:**

Based on the amount of character support contributed by EST data which make up a majority of the nuclear data set, and the lack of conflict of the nuclear data set with the simultaneous analysis tree, we conclude that the inclusion of EST data does provide a viable and efficient approach to address phylogenetic questions within a parsimony framework on a genomic scale, if problems of orthology determination and potential sequencing errors can be overcome. In addition, approaches that examine conflict and support in a simultaneous analysis framework allow for a more precise understanding of the evolutionary history of individual process partitions and may be a novel way to understand functional aspects of different kinds of cellular classes of gene products.

## Background

### Higher order Spermatophyte phylogeny: an unresolved systematics problem

**Figure 1:**
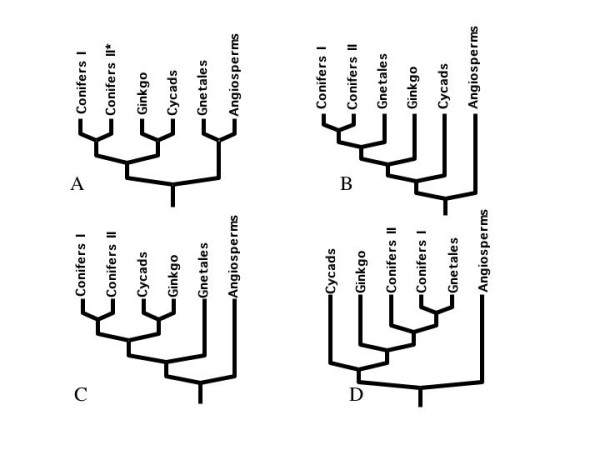
**Conflicting Phylogenetic Hypotheses involving the seed plants**. Morphological evidence (synapomophic characterstics shared between angiosperms and Gnetales) have shaped the anthophyte theory, where these two taxa form sister groups (Panel A; [1, 2, 5]). In contrast, most molecular studies postulate gymnosperms as a monophyletic group sister to all angiosperms, and place the Gnetales as a sister group to the conifers (Panels B and D; [7–9, 15, 16]). Adding to the controversy, a recent study involving phytochrome genes ([13]; Panel D) has placed the Gnetales as basal gymnosperms, with *Ginkgo *and cycads as sister taxa branching after the Coniferales.

**Figure 2:**
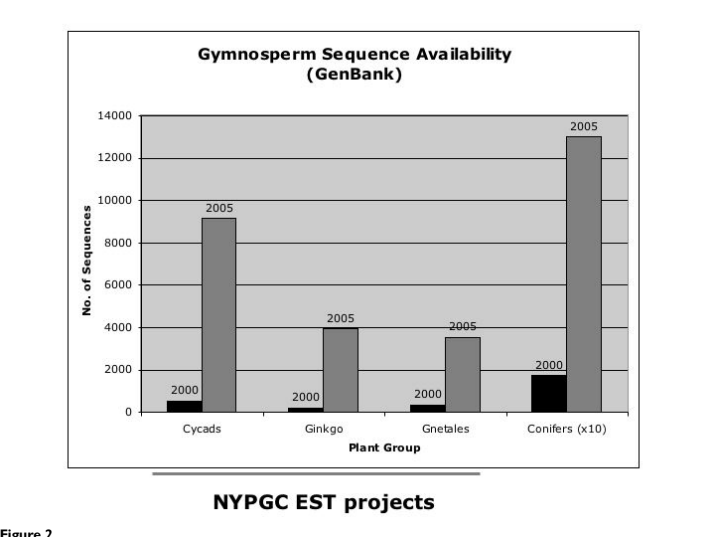
**Recent surge in gymnosperm sequence availability**. Increase in number of available nucleotide sequences in the last five years. Contributions from EST sequencing projects at the New York Plant Genomics Consortium (NYPGC), involving *Cycas rumphii, Ginkgo biloba *and *Gnetum gnemon *are reflected in these GenBank numbers.

In this paper, we discuss the utility of incorporating EST data to address one of the more important plant phylogenetic questions concerning the hierarchical relationships of the several major seed plant lineages (angiosperms, Cycadales, Gingkoales, Gnetales and Coniferales). Phylogenetic relationships among seed plant groups have remained controversial, despite attempts to resolve Spermatophyte phylogeny using numerous character sources, both morphological [1-6] and molecular [7-13]. There is a wide range of phylogenetic hypotheses that have been put forward in answer to this systematic question (see Fig. 1). Conflicting results in datasets from different sources have added to the problem. Based on morphological evidence, synapomophic characterstics shared between angiosperms and Gnetales have shaped the anthophyte theory, in which Gnetales form a sister group to the angiosperms (Fig. 1A; [1,2,5]). These synapomorphies include the presence of vessel elements, double fertilization, a double integument, and a reduction or loss of archegonia [14].

In contrast, the majority of molecular phylogenies have postulated the gymnosperms to be a monophyletic group sister to all angiosperms. Most molecular studies place the Gnetales as a sister group to the conifers (Fig. 1B; [7-9,15,16]). However, some molecular evidence can also be interpreted as supporting the anthophyte theory [17]. Attempts to associate molecular expression data with morphological structures (e.g. [18]) also place the Gnetales and conifers together, with shared expression of orthologous genes indicating that the *Gnetum *strobilar collar and ovule are homologous to the conifer bract-and-ovule/ovuliferous scale complex. Adding to the controversy, a recent study involving phytochrome genes (Fig. 1D; [13]) has placed the Gnetales as basal gymnosperms, with Ginkgoales and Cycadales as sister groups branching after the Coniferales. No recent combined analyses of molecular and morphological data have been produced and a very early one was equivocal [19]. In the past this question has been addressed with a single partition and more recently with eight [16] and thirteen partitions [20]. To our knowledge as of yet no general consensus has been reached as to the phylogenetic arrangement of these six major seed plant lineages. In fact the Tree of Life website for Spermatophytes [22] resolves only two nodes involving the five relevant taxa listed above. In the tree of life study, Gnetales are shown as the sister group to angiosperms, yet the difference between the Gnetales as the sister group to the angiosperms versus a monophyletic gymnoperms with cycads sister to other gymnosperms requires a very different set of morphological concepts and transformations. For example, are carpels leaves with marginal ovules or are they subtending leaves with axillary ovules? These are very different and mutually exclusive scenarios. Consequently, attempts to understand the genes involved in the innovations achieved by the angiosperms are severely hampered.

It is clear from the literature on seed plant phylogenetics that the addition of information relevant to the seed plants may be a viable way to solve this difficult problem. In addition, many studies on other taxa have demonstrated that the simultaneous analysis of multiple data partitions can result in an increase in overall branch support, despite conflict among the characters, due to emergent properties not evident in the separate analyses of individual data partitions [23-27]. An additional positive aspect of adding process partitions to an analysis is that once a large number of partitions from various cellular functional classes are available, partitioned analysis will also allow detailed examination of the evolutionary dynamics of these classes of genes. The latter advantage may shed light on the role of certain genes in organismal evolution.

### The (phylogenetic) trouble with ESTs

Genome level analyses have expanded our view of phylogenetics in many areas of the tree of life. With the production of whole genome DNA sequences of several taxa and large-scale EST databases as well as the incorporation of other genome enhanced technologies [27-30], a large number of candidate genes for inclusion into phylogenetic analysis have become available. In this report we utilize genome databases and explore the utility of including data from several new EST studies to increase the number of process partitions that can be used to address this difficult question in plant phylogenetics, as well as others regarding seed plant evolutionary relationships. While a number of plant genomes have been fully sequenced in recent years (*Arabidopsis thaliana*, *Oryza sativa *and *Populus trichocarpa*), limited time and resources make it difficult to sequence genomes of every living species. In some cases where genome size is very large (as is the case for most gymnosperms), the task becomes extremely impractical. A viable alternative is to sample the genome of such species through EST sequencing [31]. A number of EST sequencing projects, many in species from the more basal seed plant groups (e.g. [32-38]), have significantly increased the amount of available seed plant sequences in recent years (Fig 2).

Sequencing errors and orthology determination pose challenges to the use of ESTs as a source of characters for systematics. There can be a high rate of sequencing error in raw EST data, since it is derived from single pass reads. A strategy to minimize this problem is contig assembly and EST clustering using several reads at every region (e.g. [39,40]). In our approach, a minimum of 10 reads were used to determine each EST sequence. While orthology assessment is difficult in sub-genomic studies such as ones that use PCR or gene cloning approaches to obtain sequences, one can enhance orthology assessment in such studies by careful design of primers, and by referring to the whole genome sequences of closely related model taxa as guides for assessing orthology. Assessing orthology of ESTs is more difficult, because of the inaccuracy that accompanies EST analysis and by the possibility that some desired orthologs are not expressed or expressed at low levels. We determined the orthology of EST sequences using a tree-building approach. Initially this was accomplished by including ESTs in the gene tree analysis for each gene family. Without automation, this approach would be prohibitively time consuming and labor intensive and would greatly restrict the use of genomic-scale EST data in phylogenetic analyses. Therefore, during the course of this study, we developed automated methods for orthology determination within a parsimony framework, described elsewhere [41] (see also Methods section below for an overview).

### Why partition? Hidden support and phylogenetic inference

Studies with large numbers of process partitions in a dataset exist [23,26,42-48], and some of these have attempted to address higher phylogenetic questions of mammals, yeast and bacteria by taking advantage of genomic level approaches. These large data set approaches can be divided into whole-genome approaches (mostly microbial) and "subgenomic" [49] approaches. The whole-genome approach has the obvious advantage that orthology assessment is made with more certainty and ease when whole genome sequences are used in a phylogenetic analysis. Such is the case in a study of the relationships of seven ingroup yeast species with whole genomes sequenced [26] and much of the whole genome bacterial phylogenetic studies that are beginning to appear in the literature [44,45]. The yeast study is particularly interesting in that the authors approached the very question of where in sequence analysis space we need to be to resolve phylogenies with robustness, Using 106 carefully chosen orthologous genes they showed that > 20 genes or > 15 kb of sequence produced a plateau of robustness at measures of 100% for conventionally used detectors of node robustness (bootstrapping; [50]) in phylogenetics. In addition, they showed that despite rampant incongruence (as typified by the large number of single gene trees that disagree in topology amongst the 106 single gene trees that can be produced), combining gene partitions into a concatenated or simultaneous analysis [51,52] was always the best way to analyze the sequence information in a phylogenetic context.

Implementing a different node support measure [23] than the ones used in the yeast study, DeSalle [53] demonstrated that this phenomenon is the result of hidden support in the various gene partitions included in the analysis. Hidden support [23] is simply the amount of support at a node that is NOT found in the separate gene partitions analyzed individually. All character partitions have either positive, neutral or negative latent support for any given phylogenetic hypothesis, that becomes evident only after combining or concatenating data partitions and performing a simultaneous analysis of all available data. An assessment of hidden support using the yeast dataset of Rokas *et al*. [26] reveals that one in every five characters that support the simultaneous analysis (SA) tree is hidden [53]. This large amount of hidden support for the nodes in the SA tree, suggests that interaction of character information is an important concept in reconstructing phylogenetic relationships. More importantly quantifying hidden support can enlighten researchers about the degree of positive or negative interaction of characters in a concatenated analysis that can help determine "next steps" in phylogenetic studies. Hidden support and partitioned analyses can also aid in determining the effects of missing data and the congruence of particular partitions with an overall phylogenetic hypothesis. Issues arising from missing data are especially problematic with EST phylogenetic studies and are caused by two factors. First, in EST studies partial gene sequences are more frequently reported than full length cDNA sequences; and second, because of the random nature of clones in EST libraries often times orthologs are not found in all taxa in the study. An exploration of the amount of support contributed by each partition to the simultaneous analysis tree can aid in determining the effect of these two kinds of missing data on overall phylogenetic hypotheses.

## Results

### Phylogenetic analyses and support

**Figure 3:**
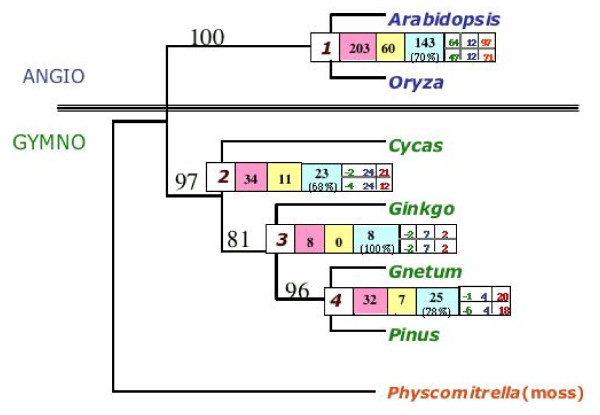
**Simultaneous analysis (SA) tree of 43 data partitions**. Single Most Parsimonious tree of the Spermatophyta, generated through the simultaneous analysis of 42 gene partitions, plus a morphological partition. Unboxed numbers on branches are bootstrap values. Node numbers are in white boxes. All other values are Partitioned Branch Support (PBS) values, as follows: Pink = total branch support (PBS); Yellow = apparent branch support (BS); Blue = hidden support (PHBS) with % total BS given below the PHBS. Branch support analysis for the cellular compartments is given to the right of the total support measures. Small boxes on right side of node indicate annotation of the total (on top) and hidden (on bottom) branch support for the three major cellular compartments. From left to right; Green = chloroplast; Blue = mitochondrion; Red = nuclear. Bootstrap values (2000 replicates) are shown on each node. The matrix has 15325 characters, 1085 phylogenetically (parsimony) informative. Tree Length: 6899 (exhaustive search); CI [90]: 0.918 (CI excluding uninformative characters: 0.739), RI [91]: 0.566.

We constructed a matrix composed of 42 gene regions consisting of mitochondrial (6), chloroplast (16) and nuclear (20, including 19 ESTs) protein and DNA (18S rDNA) sequences. In addition, we included a morphological partition with 167 characters scored for the taxa in our study. The morphological matrix was developed for this study by coding morphological characters for *Physcomitrella *but otherwise follows the morphological matrix used in previous studies [3,5,54]. A list of all the gene regions, and the accession numbers of all sequences used in the analysis, [see Additional file 1], the matrix used in the analysis [see Additional file 2] and a list of the morphological characters and character-state names [see Additional file 2] are provided. A phylogenetic analysis of the combined multiple data partitions using exhaustive tree searches resulted in a single most parsimonious tree. Figure 3 shows the result of phylogenetic analysis of these six seed plant ingroups rooted with *Physcomitrella*. This hypothesis of relationships, showing gymnosperms as a monophyletic group sister to the angiosperms, is also notable in the branching order of relationships within the gymnosperms.

In our analysis, cycads appear basal to a grouping of *Ginkgo*, *Gnetum *and conifers, with Gnetales sister to the Coniferales. This arrangement is in accordance with other recent molecular studies, that conflict with the Anthophyte hypothesis. These other studies did not include a morphological component. Our separate analysis of morphological characters supported the grouping of Gnetales with angiosperms. However, the inclusion of the morphological data set in a combined analysis contributed nine steps of hidden support to the grouping of Gnetales and Coniferales.

Total branch support on the simultaneous analysis tree in Figure 3 is 275 steps. Of those 275 steps, only 81 are apparent in the separately analyzed partitions, while 194 are hidden. In other words, 70.5% of the phylogenetically informative characters provide hidden support that would not have been apparent had each gene region been analyzed separately. Figure 3 shows the distribution of this hidden support (contributed by the 43 partitions) at each node of the tree. Strikingly, 100% of the support for node 3 is hidden (69.3% hidden for node 1, 65.6% hidden for node 2 and 78.1% hidden for node 4).

**Figure 4:**
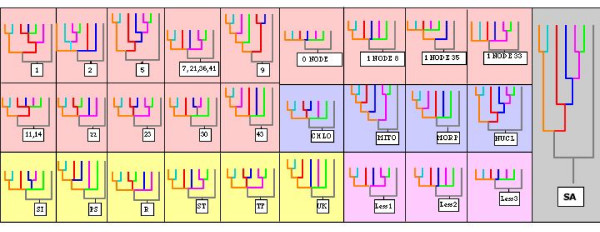
**Topological incongruence of individual process partitions with the SA tree**. The figure is a summary of bootstrap consensus trees (> 75% support) of individual (42 genes + morphology on red background) and broader (cell compartments on blue background; functional categories on yellow background) data partitions. Wide topological disagreement exists, as only one of the individual gene partitions (5, ribosomal protein S7) agrees with the simultaneous analysis (SA) tree. In broader partitions, only the nuclear data analysed together agrees with the SA tree at all nodes. Numbers in boxes indicate data partitions (see Additional File 5) represented by a particular tree. The "0 node" tree was obtained for 26 partitions (3, 4, 6, 10, 12, 13, 15, 16, 17, 18, 19, 20, 24, 25, 26, 27, 28, 29, 31, 32, 34, 37, 40, 42). Abbreviations for functional partitions (yellow background) are as follows: SI:Signalling, PI:Photosynthesis, R: Respiration, ST:Structural, TF:Transcription Factors, UK:Unknown Function; 'LessN' (on pink background) indicates trees where process partitions for one, two and three taxa are missing in the alignment. Colors of taxa in the tree are as follows; orange = *Arabidopsis*, aqua = *Oryza*, red = *Cycas*, blue = *Ginkgo*, purple = *Gnetum*, green = *Pinus*, grey = outgroup *Physcomitrella*).

**Figure 5:**
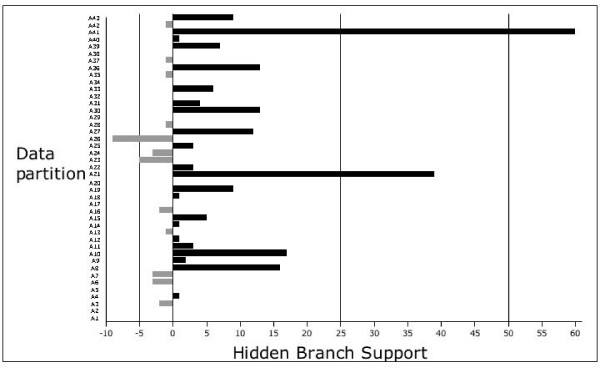
**Hidden support from individual data partitions**. This histogram shows the amount of total hidden branch support for the various process partitions examined in this study. Black bars indicate positive hidden support and fray bars indicate negative hidden support. A key to all of the partition abbreviations are given in Additional file 5.

Figure 4 shows the various strategies we used for partitioned analyses in this study. In addition to exploring the relative contribution of each individual partition to support on the tree, we also separated the sequence data into three major partitions – chloroplast (16 genes), mitochondrial (6 genes) and nuclear (20 genes). As with the yeast study [26], there is rampant disagreement amongst the 43 individual partitions–both when compared to each other, and to the combined (SA) tree (see Figure 4). But, as shown in Figure 3, there is also a large amount of hidden support in the various partitions. Nodes 2 and 3 are recovered in individual analysis of only two and four gene partitions, respectively. This observation means that, when analyzed separately, 40 and 38 gene partitions, respectively, would disagree with nodes 2 and 3, which are recovered in the simultaneous analysis tree through hidden support. Figure 5 shows the total amount of hidden branch support for each of the process partitions examined in this study.

### Effect of different partitioning strategies on hidden support

**Figure 6:**
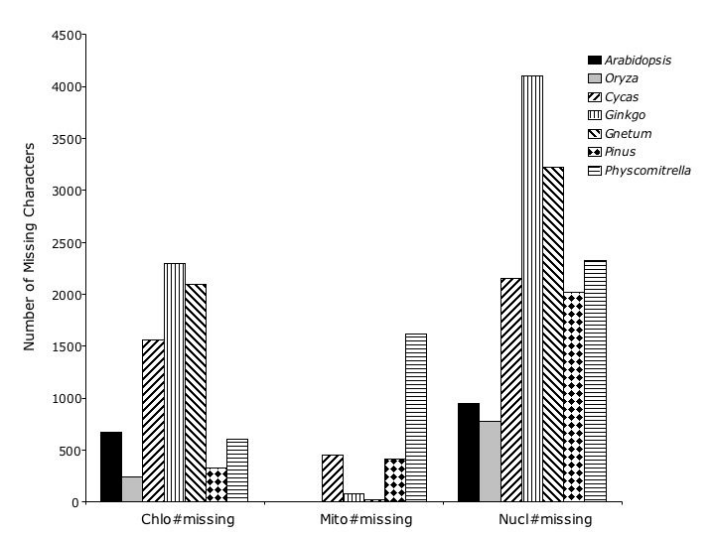
Amount of missing data for each taxon for each of the broader scale groupings of the data (chloroplast, mitochondria and nuclear).

We also examined broader groupings of the data in order to explore the effect of different partitioning schema on apparent and hidden support (Figure 3). The 20 nuclear gene partitions, when combined into a single partition, give the simultaneous analysis tree; the 16 chloroplast partitions when combined, give a tree that while not fully resolved, conflicts only at a single node with the simultaneous analysis tree; and the six mitochondrial genes placed into a single partition gave a tree with two of the four nodes in the simultaneous analysis tree that are in agreement. Figure 3 also shows the hidden branch support for the three larger partitions (chloroplast, mitochondria and nuclear). An interesting result of this method of partitioning is that it shows that the nuclear partition is the only partition of the three major ones that positively contributes branch support and hidden support to all four nodes. This is despite the fact that much of the nuclear data consist of ESTs, which are often short fragments with large amounts of missing data. One possible explanation for this result could be that the sheer number of characters in the nuclear partition swamps out the characters in the other two partitions. This does not seem to be the case, at least with the present set of genes in the nuclear, chloroplast and mitochondrial character partitions, given that the three partitions hold roughly equal numbers of raw and phylogenetically informative characters. Chloroplast and mitochondrial gene regions combined have 7268 characters, of which 478 are phylogenetically informative, while the combined nuclear gene regions have 7890 characters, of which 517 are phylogenetically informative. In addition, there is a large amount of missing data within the nuclear partition, due to the inclusion of short EST reads. Figure 6 [see also Additional file 3] shows the amount of missing data for each taxon for each of the broader scale groupings of the data (chloroplast, mitochondrial and nuclear).

In general, the broad nuclear gene data partition proved to be the most consistent with the simultaneous analysis tree. Not only is the nuclear gene tree topologically identical to the simultaneous analysis tree, but all simultaneous analysis tree nodes also receive positive branch support, both hidden and apparent, from the nuclear data set. While this might be seen as an argument for the preferential use of nuclear genes (or ESTs) in phylogenetic analyses, over the molecular characters from other subcellular compartments (such as chloroplast and mitochondria), these subcellular compartments did contribute character support to the simultaneous analysis tree despite topological conflict. In addition, the topological differences among subcellular gene partitions examined using the ILD test (implemented in PAUP*[55]) were not significant (p-value > 0.05, see below).

### Exploration of incongruence among data partitions

In order to explore the interaction among data partitions analyzed separately, we calculated ILDs [56] and tested the significance of the resulting length differences [57] between all possible pairwise comparisons of the individual data partitions as well as among the broader scale groupings of the data that we examined for hidden support above. Of the 937 pairwise comparisons, 83 showed significant length differences [see Additional file 4]. Of those 83 conflicting pairwise comparisons, 13 were comparisons between the morphological data set and an individual gene partition; 18 were between mitochondrial CO1 and another individual partition; 11 were between the nuclear heat shock protein 82 and other individual partitions; and 8 were between the chloroplast RNA polymerase beta subunit 1 and other individual partitions. We highlight these examples to show that no single partition dominated in terms of contributing conflict, but only a handful of partitions are involved in significant length differences. As with our examination of hidden support, when we examined conflict among broader scale groupings of the data, we found less conflict (as measured by ILD). In addition, none of the broader scale groupings examined for hidden support showed significant conflict (as measured by ILD) except for those groupings compared to the morphological data set.

### Effect of missing taxa

Several of the partitions we used had missing taxa due, for example, to the lack of available sequence data for a given taxon for a particular gene region [see Additional Files 1 and 3]. We explored the effect these missing taxa had on the overall phylogenetic hypothesis by comparing the amount of branch support and hidden branch support for each node using partitions where information was available for 7, 6, 5 and 4 taxa. This type of analysis is particularly relevant to EST studies as the probability of obtaining a full complement of taxa for a particular ortholog is reduced as the number of taxa in the analysis increases. Recent studies using large data sets, also containing ESTs [58,59] examined the effect of missing data by removing taxa with large amounts of missing data and comparing those results to an analysis in which these taxa were not excluded. Since the results of these analyses were similar, it was concluded that the use of taxa with large amounts of missing data did not bias the results. A simulation study [60] concluded that it is not the amount of missing data that is problematic in terms of resolving trees but the presence of too few characters to allow taxon placement.

In our analyses, we established a matrix with six ingroup taxa and one outgroup, and no taxa were removed from the matrix at any time in our analysis regardless of amounts of missing data in the various partitions. This approach allowed us to explore the effect of the inclusion of taxa with missing data by examining branch support values contributed to the simultaneous analysis tree by partitions with varying amounts of missing data. In this case, we compared the contribution to support provided by those partitions that contained at least some data (but not necessarily the complete dataset) for all seven taxa to the group of partitions that were lacking data for one taxon for an entire partition (an individual gene region); to those that were lacking data for two taxa and so forth. In this way we were able to examine the affect of incompletely taxonomically sampled partitions.

**Figure 7:**
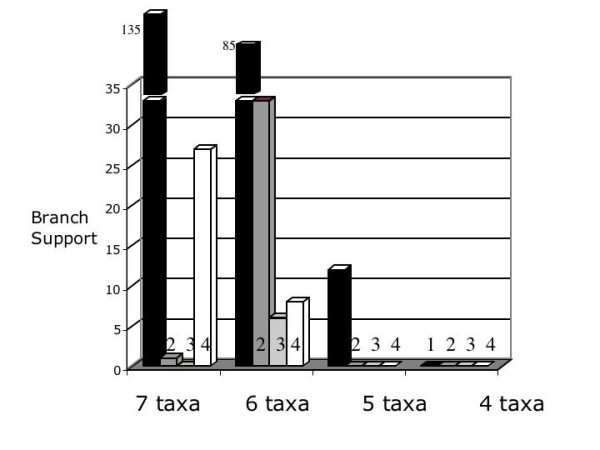
**Effect of missing data on branch support**. The randomness of EST sequencing results in some partitions not being found for all taxa. This histogram shows the effect of missing taxa in process partitions on support for each node. Support decreases as less taxa are included in the analysis however, these results do not take into account the varied amount and distribution of data available for each taxon (see text). The shading of the bars indicates the node in the SA tree: black = node 1; darker gray = node 2; lighter gray = node 3 and white = node 4. Branch support is given on the Y-axis, and the number of taxa in a process partition are given in clusters on the X-axis.

The result of our analysis is shown in Figure 7. While both branch support [21,61,62] and hidden support [23] drop to zero when there are fewer than 6 taxa in a partition, suggesting that individual partitions lacking more than two taxa do not contribute support to the tree, there were differences in the overall amount and distribution of data for each taxon; making a direct correlation to taxon number difficult to establish with the present data set. The character set containing partitions that had at least some data for all seven taxa (partitions: A1, A2, A3, A5, A7, A9, A10, A11, A12, A14, A15, A24, A25, A26, A30, A31, A32, A34, A35, A36 and A43) consisted of 8399 characters of which 544 were parsimony informative. The character set consisting of partitions that had at least some data for six taxa (partitions: A6, A8, A13, A16, A18, A21, A22, A23, A27, A28, A33, A39, A40, A41 and A42) consisted of 5310 characters of which 482 characters were parsimony informative. The character set consisting of partitions that had at least some data for five taxa (partitions: A4, A19, A20 and A37) consisted of 767 characters of which 48 characters were parsimony informative. The character set consisting of partitions that had at least some data for four taxa (partitions: A17, A29 and A38) consisted of 849 characters of which 11 characters were parsimony informative.

### Effect of different functional classes of genes

We also partitioned the data set into classes of genes based on their cellular function. Our functional partitions were MnoPSR (Non-Photosynthetic or Respiratory Metabolism: 7 gene partitions), photosynthetic (11 gene partitions), respiration (7 gene partitions), signalling (3 gene partitions), structural (8 gene partitions), transcription factors (2 gene partitions) and genes of unknown function (4 gene partitions).

**Figure 8:**
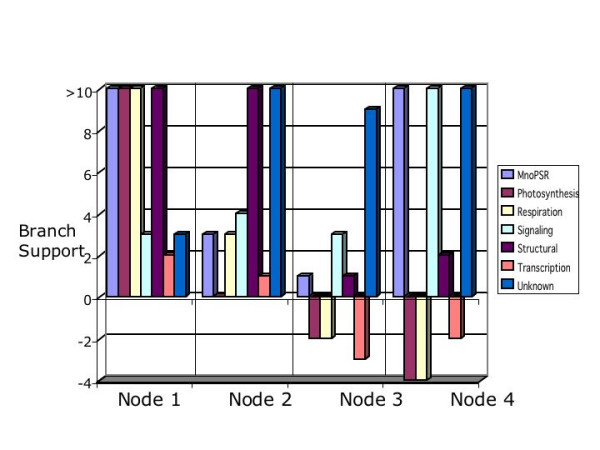
**Node support from functional partitions**. Support for each node in the simultaneous analysis tree from the various cellular function process partitions. The cellular function partitions are given in the legend to figure 4. The Y-axis indicates the branch support value. Node information is in clusters of process partitions as indicated on the X-axis. For simplicity, branch support values larger than ten are depicted here as 10.

Figure 8 shows the results of this partitioning exercise where the raw support for a node is depicted as a function of the different classes of genes. When the data are partitioned in this fashion, nodes 3 and 4 can be characterized as having negative branch supports for some of the classes of genes. Nodes 1 and 2 are supported by all functional classes of genes. One of the more striking results in this figure is the negative branch support obtained from transcription factors, respiration genes and photosynthetic genes. Alternatively, positive support for all four nodes is obtained from the metabolic genes, structural genes, signalling proteins and proteins of unknown function.

While our results would suggest that conserved structural proteins and signalling proteins might be better at defining both deeper nodes and tree tip nodes, and proteins in the transcription factor and respiration class of genes might be best for nodes nearer the tips of the tree; the sample size of genes in functional partitions for this study is small. Nevertheless, these results do suggest a potential method for categorizing functional gene classes with respect to their congruence with a simultaneous or organismal phylogeny. As more ESTs are added to the sequence database, the sample sizes of these functional classes will become larger and a more rigorous test of the role of functional class in phylogenetic analysis may be possible.

## Discussion

### Where to from here?

Rokas *et al*., [26] addressed the question of where in sequence analysis space we need to be to robustly resolve phylogenies; showing that > 20 genes or > 15 kb of sequence produced a plateau of robustness at measures of 100% for conventionally used detectors of node robustness. The > 15, 000 base pairs and > 40 genes in the present study is only enough to garner strong support for three of the four nodes in the concatenated analysis tree with node 3 receiving the weakest support in the analysis. More sequence information is thus needed to resolve this problem, and it appears from the hidden support analysis that nuclear gene partitions will most efficiently provide information for all nodes in the concatenated analysis tree. In addition, both the yeast analysis by Rokas *et al*. [26], and the seed plant analyses presented here strongly suggest that even though a single gene partition might support an alternative topology to the concatenated analysis tree, hidden support in most gene partitions will contribute positively to overall robustness of a phylogenetic hypothesis. Finally, the yeast and seed plant examples, while having similar numbers of ingroup taxa, suggest that different numbers of characters and genes will be needed to assign robust inferences to nodes in studies. We suggest that this discrepancy may be a factor of the different phylogenetic ages of the groups: the ingroup species in the yeast phylogeny diverged between 50 and 100 MYA [63,64] is basically within a genus, while the ingroup taxa in the plant study diverged no earlier than 400 MYA [65-67]. In addition, several studies with much larger numbers of ingroup taxa exist [23,42,44] and these studies suggest that larger numbers of characters than those of the yeast study are required for robust resolution of this simple phylogenetic hypothesis. How many more characters? A strong indication may be given by the high support and robustness of node 1 (*Arabidopsis *+ *Oryza*). A plateau of topological robustness for other nodes may be reached when a similar number of phylogenetically informative characters is reached.

The approach we describe here, where support for the SA tree is estimated for each process partition, will also pinpoint those partitions that disagree or conflict with the overall general pattern of divergence of the taxa in the analysis. If one assumes that the SA tree best represents evolutionary history of the taxa involved, then such partitions are in conflict with overall organismal history of the taxa in the analysis. This approach then would provide a method for detecting process partitions that might be selected for or have experienced drift and such partitions might be important in some of the more interesting organismal differences amongst the taxa in the analysis.

One final and important aspect of the present analysis highlights a problem that will be prevalent in future genomic level phylogenetic studies. This problem concerns the almost continual revision of the overall phylogenetic hypothesis for a set of taxa. For instance, as more and more EST data are added to the database, more and more process partitions can be added to an analysis. This will effectively create a growing matrix that might even expand daily. With the addition of each new process partition to an analysis, all support values and other tree metrics such as bootstrap values [50], jackknife values [68,69], Bayesian posterior probabilities [70-72], and node support values [21-23,42,61,62,73] need to be recalculated. In addition, the manual inclusion of the new process partitions to a growing matrix is time consuming and sometimes prone to error. We therefore suggest that such important systematic questions where large amounts of genomic level data are available have a need for an automated and rapid means for inclusion of new process partitions to the growing matrix. Such an automated approach is under development for the seed plant question and will be discussed in a separate publication [74].

## Conclusion

• Simultaneous analysis using 42 gene partitions and a morphological partition yield a phylogenetic hypothesis with a monophyletic gymnosperms which is at odds with the Anthophyte hypothesis.

• Addition of short EST sequences to a data set can enhance a phylogenetic analysis, if the problems of sequence quality and orthology are overcome.

• The majority of support in this study is hidden support, meaning that the support is not immediately apparent in single gene partitions.

• Completeness of data partitions with respect to full complement of taxa had a large affect on levels of support in phylogenetic analysis. In our study example with seven taxa, support from partitions that had sequences for five or fewer taxa was nonexistent. However, variation in the amount and distribution of data within partitions may also play a role.

• When phylogenetic incongruence between a partitioned functional class of genes (such as transcription factors) and the organismal phylogeny is detected, this result suggests that the partition has experienced a unique evolutionary history relative to the organisms. This different evolutionary history can be used as a signpost of altered evolutionary pressure in a particular class of genes. In this way, incongruence of a particular class of genes (such as transcription factors) in a partitioned analysis allow us to establish hypotheses about the evolution and potential function of these gene classes.

## Methods

### Orthology determination and phylogentic analyses

Many studies use pairwise sequence comparison schemes, such as BLAST [75], COG (Clusters of Orthologous Groups; [76]), INPARANOID [77,78], RBH (Reciprocal Blast Hits; [79,80]), and RSD (Reciprocal Smallest Distance Algorithm; [81]) to determine gene orthology on a genomic scale.

Since we are ultimately interested in exploring the characters associated with particular evolutionary novelties, we use a character-based alternative to distance based methods for the identification of orthologous gene regions. The tree-building approach to orthology determination involves the generation of gene family trees in order to identify the orthologous gene family member for each EST sequence. Within a character-based parsimony framework, nodes are defined by shared derived characters.

Without automation, this approach would be prohibitively time consuming and labor intensive and would greatly restrict the use of genomic-scale EST data in parsimony based analyses since the placement of ESTs into orthology groups using this character-based approach would require manual rebuilding of gene family trees for each new EST to be classified. Therefore, during the course of this study, we developed automated methods for orthology determination within a parsimony framework: OrthologID [41], [92] firefox/Netscape is the preferred browser–Internet Explorer is not supported by the current OrthologID viewer). This approach builds gene family trees using sequences from available completely sequenced genomes (currently, *Arabidopsis*,*Oryza *and *Populus; Chlamydomonas reinhardtii *is used as an outgroup in the gene tree analyses for orthology determination), such that all members of a given gene family are included. In addition, rather than including EST sequences in the gene tree construction analysis, the whole genome gene family trees are first used to construct "guide" trees. These gene family guide trees are used to identify diagnostic characters for each gene family member and then EST query sequences are screened for the presence of shared diagnostics using the CAOS algorithm (See [82] for details of the guide tree/CAOS approach). This approach eliminates the need to manually rebuild a gene family tree each time a new EST sequence requires orthology determination.

We use only completely sequenced genomes for constructing gene family guide trees with OrthologID in order to minimize the possibility of the erroneous placement of query sequences due to missing data. If gene family guide trees had been constructed using partially sequenced genomes, it is possible that some gene family members would be missing, in which case it could be possible that queries orthologous to these missing gene family members would be incorrectly placed. The current database of plant genomes will soon be expanded to include complete genomes from other phylogenetic lineages, including prokaryotes and non-plant eukaryotes.

OrthologID automatically searches the local database of completely sequenced plant genomes and performs an initial clustering of gene sequences into putative gene families, using NCBI BLAST [83] with an expectation value cutoff of 1e^-20^. Next it builds gene family trees. It performs sequence alignments using the program MAFFT [84] using different sets of alignment parameters to create three different alignments for each gene family and culls [85] alignment ambiguous regions. The three pairs of gap open penalty and offset values are (1.53, 0.123), (2.4, 0.1), and (1.0, 0.2). It performs tree searches within a parsimony framework, using either exhaustive searches or, where exhaustive tree searches are not possible due to a large number of putative gene family members, heuristic searches are performed implementing the parsimony ratchet [86] with 200 re-weighting iterations for each of 20 ratchets; in order to rigorously explore tree space. It saves resultant trees and computes the strict consensus when multiple equally parsimonious trees are obtained from the analysis and then passes these guide trees to the CAOS algorithm to identify node diagnostics. In order to identify the ortholog of an EST sequence, OrthologID uses the CAOS algorithm to screen the ESTs for the presence of characters that are diagnostic of nodes on the guide tree.

Once EST orthologs had been identified, we manually assembled a process partition matrix for each orthologous gene region for all of the seven chosen plant taxa; sequences for the moss *Physcomitrella patens *[87,88] were used as outgroup in all phylogenetic analyses. We aligned the sequences for each process partition using the default parameters in Clustal [89]. We assembled a simultaneous analysis matrix composed of 42 gene regions consisting of mitochondrial (6), chloroplast (16) and nuclear (20; including 19 composed of EST protein sequences and one of DNA sequences [18S rDNA]) along with a single morphological partition. A list of all the gene regions, and the accession numbers of all sequences used in the analysis, can be found in Additional file 1. In several instances in which a mitochondrial or chloroplast sequence was not available for a given taxon, we substituted the corresponding sequence from a related species. These are noted inAdditional file 1. The matrix used in the analyses can be found as Additional file 2. Phylogenetic analyses were accomplished in PAUP* version 4.0b1.0 [55] using exhaustive searches. Measures of branch support [21,23,61,62] were accomplished using batch command files in PAUP*, and the resulting log files were imported into an Excel spreadsheet for final calculations.

## Authors' contributions

MGE and JEBdlT were responsible for the primary collection of information from the EST databases and for the construction of the phylogenetic matrix. EDB and JEBdlT generated many of the EST sequences for *Cycas rumphii, Ginkgo biloba *and *Gnetum gnemon *and other species in the New York Plant Genomics Consortium (NYPGC). MK was responsible for programming the scripts used to extract sequences from the EST databases. RD, MGE and JEBdlT were responsible for the partitioned data analyses and their interpretation. DWS was responsible for the morphological data set and DWS and GMC were responsible for interpretation of results in a plant phylogenetics and evolutionary context.

## Supplementary Material

Additional File 1 **Accession numbers for sequences used in the analysis**. Accession numbers, by partition name, used to assemble the data matrix. Database of origin is indicated before each number. In some cases, more than one EST was used (i.e. clustered) to assemble a single partition. Click here for file

Additional File 2 Table 2 – Data matrix used for analyses in this paper in NEXUS format. This file contains the raw data matrix of characters used in the phylogenetic analysis. Click here for file

Additional File 3 **Table 3 – Proportion of missing characters per taxon and data partition. **This file lists the proportion of missing characters used in the analysis. Click here for file

Additional File 4 **Table 4 – Pairwise analysis of congruence among individual partitions**. Table showing significance scores when the Incongruence Length Difference (ILD) test was applied to pairwise comparisons among each individual partition. Statistically significant numbers (i.e. equal or smaller than 0.05), showing phylogenetic incongruence among partitions, are shaded. Click here for file

Additional File 5 Table 5 – Key to partition names. Click here for file
