# Supplementary material for: ESTimating plant phylogeny: lessons from partitioning
Source: BMC Evol Biol. 2006 Jun 15;6:48. doi: 10.1186/1471-2148-6-48 (PMC1564041; doi:10.1186/1471-2148-6-48)
Supplement: Additional File 3 — Table 3 – Proportion of missing characters per taxon and data partition. This file lists the proportion of missing characters used in the analysis. [file 1471-2148-6-48-S3.pdf]

Table 3: Proportion of missing characters per taxon and data partition.

	Taxa							Partition Size	
	Arab.	***Oryza***	***Cycas***	***Ginkgo***	***Gnetum***	***Pinus***	***Physco.***	**Separate Parts**	**Grouped Parts**
A1	0	0	10	31	34	32	0	479	
A2	69	37	77	55	79	2	2	125	
A3	0	0	0	0	16	0	0	159	
A4	0	0	81	81	0	0	0	81	
A5	0	0	0	0	0	0	0	39	
A6	0	0	41	0	0	0	0	41	
A7	0	0	0	0	0	0	0	43	
A8	0	0	33	750	258	0	0	750	
A9	0	0	10	30	310	6	4	498	
A10	548	8	548	548	548	0	0	710	
A11	40	39	14	14	21	4	0	512	
A12	2	2	38	39	38	0	0	75	
A13	8	0	8	16	28	83	0	83	
A14	0	0	26	26	29	0	0	353	
A15	0	0	55	54	54	0	0	473	
A16	2	3	274	111	136	0	53	274	**Chlo**
A38	0	148	340	538	538	198	538	538	5233
A18	0	0	21	23	4	5	57	57	
A19	0	0	368	0	0	368	0	368	
A20	0	0	53	53	0	0	0	53	
A21	0	0	0	2	11	34	670	670	
A22	0	0	0	0	0	0	471	471	**Mito**
A23	0	0	10	0	1	0	416	416	2035
A17	0	0	1	24	156	156	156	156	
A24	124	86	0	77	31	0	69	278	
A25	117	117	325	304	289	117	34	497	
A26	0	0	0	0	0	0	186	241	
A27	4	0	41	280	62	116	87	280	
A28	18	17	119	224	111	19	23	224	
A29	155	155	0	0	0	0	155	155	
A30	6	6	453	638	638	424	452	711	
A31	0	56	43	195	0	7	0	195	
A32	327	160	2	541	478	301	461	625	
A33	1	0	445	281	285	280	257	445	
A34	4	4	1	4	121	0	0	156	
A35	74	64	16	127	47	9	0	234	
A36	0	0	78	78	1	78	1	1829	
A37	0	0	265	0	265	0	0	265	
A39	0	0	0	326	35	25	0	326	
A40	109	109	357	325	603	396	349	603	
A41	0	0	0	400	92	88	88	400	**Nucl**
A42	0	0	0	270	0	0	0	270	7890
A43	0	0	0	0	0	0	0	167	**Morp**
Total # Missing Characters	1608	1011	4153	6465	5319	2748	4529	15325	
								**Matrix Size**	
Percent Missing Characters	10.5	6.6	27.1	42.2	34.7	17.9	29.6		
	Arab.	***Oryza***	***Cycas***	***Ginkgo***	***Gnetum***	***Pinus***	***Physco.***		
