# Supplementary material for: ESTimating plant phylogeny: lessons from partitioning
Source: BMC Evol Biol. 2006 Jun 15;6:48. doi: 10.1186/1471-2148-6-48 (PMC1564041; doi:10.1186/1471-2148-6-48)
Supplement: Additional File 4 — Table 4 – Pairwise analysis of congruence among individual partitions. Table showing significance scores when the Incongruence Length Difference (ILD) test was applied to pairwise comparisons among each individual partition. Statistically significant numbers (i.e. equal or smaller than 0.05), showing phylogenetic incongruence among partitions, are shaded. [file 1471-2148-6-48-S4.pdf]

Table 4: Pairwise analysis of congruence among individual partitions

	A1	A10	A11	A12	A13	A14	A15	A16	A17	A18	A19	A2	A20
A1	1												
A10	0.95	1											
A11	1	0.91	1										
A12	0.37	0.75	0.45	1									
A13	0.54	1	0.43	1	1								
A14	1	1	1	0.23	0.47	1							
A15	0.88	0.98	0.79	0.62	1	0.96	1						
A16	0.03	0.01	0.03	0.3	1	0.09	0.05	1					
A17	1	1	1	1	NA	1	1	NA	1				
A18	0.48	0.66	0.73	0.47	1	1	1	1	NA	1			
A19	0.19	1	0.16	1	1	0.26	0.16	1	NA	1	1		
A2	0.56	0.81	0.58	0.62	0.7	0.25	0.92	0.47	1	0.37	0.46	1	
A20	1	1	1	0.7	0.36	1	1	0.58	NA	1	NA	1	1
A21	0.08	0.07	0.2	0.18	0.69	1	1	1	NA	1	1	0.35	1
A22	1	0.01	0.01	0.16	1	1	0.01	1	NA	0.31	1	0.05	1
A23	0.04	0.06	0.42	0.3	0.33	1	0.19	1	NA	1	1	0.25	1
A24	0.95	0.77	0.86	0.74	0.26	1	0.9	0.18	1	0.6	0.42	0.49	1
A25	0.97	1	0.82	0.73	0.49	0.77	0.8	0.54	1	0.26	0.54	0.42	0.67
A26	0.95	0.5	0.88	1	1	1	0.9	1	1	1	1	0.67	1
A27	0.4	1	1	0.27	1	1	0.62	0.76	NA	1	1	0.38	1
A28	0.44	0.59	0.06	1	1	0.48	0.35	0.08	NA	0.05	1	0.8	0.19
A29	1	1	0.23	1	NA	1	1	NA	NA	NA	NA	0.54	NA
A3	0.45	0.4	0.27	0.98	1	0.72	0.43	0.45	1	1	1	0.65	0.28
A30	0.34	1	0.06	0.07	0.13	1	0.93	0.02	1	0.32	0.04	0.36	1
A31	0.26	0.42	0.18	1	1	0.74	0.22	0.38	NA	0.89	0.02	0.81	0.45
A32	1	1	1	1	1	1	1	1	1	1	1	1	1
A33	0.56	0.02	0.2	0.02	0.03	0.69	0.26	0.01	NA	1	0.01	0.53	1
A34	0.45	0.78	0.61	0.89	1	0.57	0.65	0.42	1	0.35	1	0.86	1
A35	0.26	0.03	0.02	0.25	0.37	0.17	0.12	0.13	1	0.45	0.54	0.12	0.71
A36	0.41	1	0.65	0.5	0.38	1	0.77	1	1	1	1	0.97	1
A37	0.74	0.24	0.58	0.48	0.16	0.5	0.68	0.3	NA	0.43	0.12	0.84	1
A38	1	1	1	1	1	1	1	1	NA	1	NA	1	NA
A39	0.18	1	1	0.67	1	1	0.82	0.32	NA	1	1	0.66	1
A4	0.59	0.56	0.37	1	1	0.24	0.59	0.36	NA	1	NA	0.35	NA
A40	0.56	0.15	0.56	0.32	1	0.72	0.46	0.08	NA	1	1	1	1
A41	0.03	1	1	0.22	1	1	0.58	0.75	NA	1	1	0.46	1
A42	0.27	0.27	0.03	1	1	0.52	0.25	0.1	NA	0.18	1	0.3	0.45
A43	0.35	0.26	0.15	0.89	0.58	0.57	0.05	0.01	1	0.57	0.01	0.51	0.23
A5	0.58	0.63	0.57	0.82	0.72	0.15	0.77	0.65	0.07	0.42	0.24	1	1
A6	0.62	0.59	0.56	1	0.35	0.29	0.56	0.62	NA	0.55	0.33	0.55	1
A7	0.23	0.67	0.62	0.72	1	1	0.35	0.36	1	1	0.39	0.63	1
A8	0.1	0.47	0.22	0.7	1	0.24	0.45	0.44	NA	1	1	0.39	1
A9	0.18	0.05	0.14	0.63	0.52	0.14	0.04	0.05	1	0.44	0.22	0.24	0.31

Table 4: Pairwise analysis of congruence among individual partitions (continued)

	A21	A22	A23	A24	A25	A26	A27	A28	A29	A3	A30	A31	A32
A21	1												
A22	0.01	1											
A23	0.06	0.01	1										
A24	0.11	1	0.12	1									
A25	0.09	0.1	0.19	0.85	1								
A26	0.08	0.01	1	1	1	1							
A27	1	0.01	1	0.75	0.52	0.91	1						
A28	0.01	0.16	0.02	0.64	1	0.86	0.16	1					
A29	NA	NA	NA	0.65	1	0.04	NA	NA	1				
A3	0.3	1	1	0.86	1	1	0.24	0.82	0.69	1			
A30	0.02	0.04	0.01	1	0.71	0.05	1	0.71	1	0.04	1		
A31	0.06	1	0.17	0.65	0.86	1	0.6	0.91	NA	0.57	0.66	1	
A32	1	1	1	1	1	1	1	1	1	1	1	1	1
A33	1	1	1	0.45	0.33	1	0.52	0.13	NA	0.02	0.07	0.25	0.99
A34	0.12	0.03	0.09	0.95	0.28	0.86	0.49	0.88	1	0.96	0.48	0.93	1
A35	0.21	1	0.2	0.09	0.4	0.32	0.16	1	1	0.05	0.08	0.7	1
A36	1	0.01	0.53	0.66	0.88	1	0.07	0.02	0.23	0.16	0.37	0.06	1
A37	0.02	0.23	0.04	0.87	0.8	0.75	0.6	0.8	NA	0.62	0.16	0.83	1
A38	1	1	1	1	1	1	1	1	NA	1	1	1	1
A39	1	0.01	1	0.49	0.71	0.75	1	0.12	NA	0.25	1	0.4	1
A4	1	1	1	1	0.43	1	0.49	0.59	NA	0.34	0.4	0.4	1
A40	1	1	1	1	0.88	1	1	1	NA	0.32	0.9	1	1
A41	1	0.01	1	0.3	0.3	0.76	1	0.04	NA	0.12	1	0.18	1
A42	0.01	0.01	0.02	0.52	0.37	0.67	0.06	0.25	NA	0.19	0.7	0.13	1
A43	0.01	0.01	0.01	0.52	0.9	1	0.03	0.07	0.52	0.32	0.03	0.06	1
A5	0.5	0.28	0.3	0.63	0.17	0.73	1	1	1	0.88	0.45	1	1
A6	0.12	0.31	0.21	0.34	1	1	1	1	NA	1	0.05	1	1
A7	1	0.02	1	0.66	0.4	1	1	0.28	0.13	0.69	0.05	0.84	1
A8	1	0.01	1	0.22	0.22	0.58	0.18	0.03	NA	0.27	0.72	0.1	1
A9	0.04	0.01	0.16	0.32	0.39	1	0.31	0.85	0.18	0.27	0.03	0.87	1

	A33	A34	A35	A36	A37	A38	A39	A4	A40	A41	A42	A43	A5
A33	1												
A34	0.16	1											
A35	0.14	0.79	1										
A36	0.21	0.76	0.03	1									
A37	0.63	0.37	0.74	0.44	1								
A38	1	1	1	1	1	1							
A39	0.41	0.75	0.05	0.65	0.52	1	1						
A4	0.58	1	0.58	0.48	0.85	NA	0.38	1					
A40	0.28	0.3	1	1	0.27	1	1	1	1				
A41	0.12	0.52	0.02	0.71	0.48	1	1	1	1	1			
A42	0.13	0.55	0.1	0.01	0.51	1	0.05	1	1	0.04	1		
A43	0.01	0.75	0.1	0.01	0.21	1	0.03	0.12	0.53	0.02	0.01	1	
A5	0.39	0.72	0.23	0.79	0.56	1	1	1	0.08	1	1	0.71	1
A6	0.4	0.81	0.28	0.65	1	1	1	1	0.25	1	1	0.44	1
A7	0.14	0.7	0.05	0.78	0.22	1	1	0.27	1	1	0.29	0.3	0.79
A8	0.03	0.5	0.04	0.61	0.1	1	0.77	0.3	1	1	0.09	0.02	1
A9	0.01	0.25	0.02	0.18	0.15	1	0.5	0.33	1	0.37	0.3	0.44	0.31

	A6	A7	A8	A9
A6	1			
A7	0.36	1		
A8	1	1	1	
A9	0.1	0.43	0.4	1
