# Supplementary material for: ESTimating plant phylogeny: lessons from partitioning
Source: BMC Evol Biol. 2006 Jun 15;6:48. doi: 10.1186/1471-2148-6-48 (PMC1564041; doi:10.1186/1471-2148-6-48)
Supplement: Additional File 5 — Table 5 – Key to partition names. [file 1471-2148-6-48-S5.pdf]

Table 5: Key to Partition Names

Partition No.	Name	
A1	rbcL	
A2	ribosomal prot S12	
A3	ribosomal prot S7	
A4	Photosystem I-C	
A5	cytochrome b559Beta	
A6	Photosystem IIL 5KDa	
A7	Photosystem II-NC	
A8	Photosystem I-A	
A9	ATP synthase Beta	
A10	RNA Polymerase	
A11	Photosystem II P680	
A12	PSII Phosphoprotein subunit H	
A13	cytochrome b559 Alpha	
A14	Photosystem II-D2	
A15	Photosystem II CP43	
A16	Chlorophyll A/B binding prot	
A38	ASP Aminotransferase	**Chloroplast**
A18	NADH1	
A19	NADH5	
A20	NADH2	
A21	Maturase	
A22	Cytochrome C Oxidase I	
A23	ATPaseAlpha	**Mitochondrial**
A17	Glyceraldehyde-3-P dehydrogenase (GPDH)	
A24	40S Ribosomal S15	
A25	Actin 2/7	
A26	AGAMOUS	
A27	Catalase	
A28	Enolase	
A29	Epimerase	
A30	HSP 81-2	
A31	Glutathione Peroxidase (PHGP)	
A32	Polyubiquitin 10	
A33	Proteasome 26S	
A34	Ribosomal Protein S18	
A35	UBQ Conjugating Enzyme E2	
A36	18S rDNA	
A37	Expansin	
A39	Zn finger - DNA binding	
A40	Clathrin Coat protein	
A41	Delta-TIP tonoplast protein	
A42	ATLS1 Light-inducible prot	**Nuclear**
A43	**Morphology**	
